# Prevalence of exposure to needle stick and sharp‐related injury and status of hepatitis B vaccination among healthcare workers: A cross‐sectional study

**DOI:** 10.1002/hsr2.1399

**Published:** 2023-07-05

**Authors:** K. C. Rupak, Dipendra Khadka, Sabal Ghimire, Aayush Bist, Ishant Patel, Smriti Shahi, Natasha Dhakal, Ibeja Tiwari, Dhan B. Shrestha

**Affiliations:** ^1^ Department of Medicine Nepalgunj Medical College Banke Nepal; ^2^ Department of Medicine Lumbini Medical College Lumbini Nepal; ^3^ Department of Medicine Mount Sinai Hospital Chicago Illinois USA

**Keywords:** healthcare workers, hepatitis B, needle sticks, Nepal, sharp‐related injuries

## Abstract

**Background and Aims:**

Hepatitis B is a leading cause of chronic liver disease and subsequent liver transplantation. This is a vaccine‐preventable illness. Health workers continue to be at risk for blood‐borne pathogens due to occupational exposures. The overall goals of our study were to determine the prevalence of exposure to needle sticks and sharp‐related injuries (NSSI) and hepatitis B vaccination status among healthcare workers (HCWs) of Nepalgunj Medical College Teaching Hospital (NGMCTH), Kohalpur, Banke, Nepal.

**Methods:**

A descriptive cross‐sectional study was conducted among HCWs at the NGMCTH following ethics approval by the NGMCTH Ethics Review Committee. A pretested structured questionnaire was used to compile the data. Data was collected from September 15, 2021 to September 14, 2022. Collected data entered in Microsoft Excel and analyzed using statistical package for social sciences version 22. Analyzed data were presented using simple descriptive statistics with appropriate figures and tables.

**Results:**

A total of 304 among 506 HCWs (60.1%) participated in the survey were exposed to Needle sticks. Nine of whom (3.7%) were injured substantially (more than 10 times). Among nursing students, 21.3% had experience with NSSI. 71.7% of HCWs had received at least one dose of the hepatitis B vaccine, of whom 61.9% (44.5% of total HCWs) had received three doses.

**Conclusions:**

This study demonstrated that more than two‐quarters of HCWs were exposed to NSSI. Despite being at risk, vaccination status was still low, and less than half only received three complete doses. Precaution should be taken when working with instrumentation and procedures. Hepatitis B immunization programs for HCWs should be delivered at no cost with 100% coverage and protection. Raising awareness about hepatitis B infection and immunization remains crucial to primary prevention.

## 1 INTRODUCTION

Hepatitis B virus (HBV) is the most common blood‐borne transmissible disease seen in healthcare settings with higher endemicity in Asian countries (75%) especially South East Asia Region (10%–20%).^1^, ^2^, ^3^ The Centers for Disease Control and Prevention estimates that over 380,000 percutaneous‐related injuries occur annually among hospital employees and approximately half of such exposures go unreported. Globally, about 32.4%–44.5% of healthcare workers (HCWs) report being exposed to accidental needle‐stick and sharp‐related injuries each year. Needle sticks and sharp‐related injuries (NSSI) cause health hazards of having transmission of about 40% of HBV and HCV and 2.5% of human immunodeficiency virus (HIV)/acquired immunodeficiency syndrome globally to HCWs. Among them, 90% of these infections happened in underdeveloped and low‐income countries where standard precautions methods are very poor. Also, underreporting of NSSI injuries by HCWs is not much encouraging. Poor knowledge, awareness, and attitude toward safety precautions might be associated with such injuries.^4^, ^5^ World Health Organization (WHO) annual report states such injuries occur four per HCWs in Asia.^6^ HBV infection is a preventable disease by vaccination. The risk of transmission in nonimmunized is about 6%–30%. HBV Vaccine and gamma globulin reduce this risk by 90%–95%. But unfortunately, the WHO report shows vaccine coverage among HCWs worldwide is very low. In underdeveloped and developing countries, it is about only 18%–39%.^7^, ^8^

Though WHO has recommended special consideration for HCWs and medical students for a screening of HBV and vaccination, Nepal Government lacks a policy‐level program to vaccinate such at‐risk populations.^9^ Similarly, health institutions cannot make necessary arrangements to protect their staff and trainees.^10^ HCWs, especially nurses and laboratory staff, are the first level of contact between patients and medical care.^11^ About three million HCWs every year are exposed to pathogens, of which two million are exposed to HBV, 0.9 million to hepatitis C (HCV), and 0.1 million are exposed to HIV. Personal, legal, and Professional hazards to HCWs are the effects of needle stick injuries.^3^

Our study aims at exploring the HBV vaccine coverage among HCWs, including clinical students at Nepalgunj Medical College Teaching Hospital (NGMCTH). Additionally, the study also aimed to explore the prevalence of occupational exposure to the HBV by NSSI.

## 2 METHODS

### 2.1 Study design and setting

This descriptive cross‐sectional study was done to assess the prevalence of exposure to NSSI and the status of hepatitis B vaccination among HCWs at NGMCTH, Kohalpur, Nepal. Ethical approval was taken from NGMCTH Ethics Review Committee. Data were collected using a pretested structured questionnaire after taking informed consent from them. All participants were assured of confidentiality. The study began on September 15, 2021 to September 14, 2022, a period of 1 year. The English language was used for better response with professional health workers during data collection. Total sampling method was used and all health workers who are eligible to participate in this study were included. Potential bias may include recall bias and information bias.

### 2.2 Study participants

A total of 506 individuals including clinical Bachelor of Medicine and Bachelor of Surgery and Nursing students, laboratory staffs, and consultants/residents from different departments were taken as the sample population.

### 2.3 Eligibility criteria

**Figure 1:**
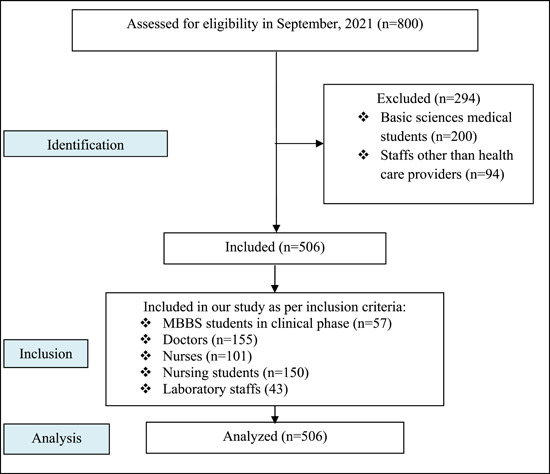
STrengthening the Reporting of OBservational studies in Epidemiology (STROBE) for observational studies flow diagram.

Students from clinical postings (medical students and nursing students), residents, nurses, consultants, and laboratory staff who are willing to participate in this study have been included. Students from basic science and other staff have been under our exclusion criteria (Figure 1).

### 2.4 Questionnaire administration

Pretested semistructured self‐administered questionnaire in printed form was administered to obtain primary data from study participants. Sociodemography details, the prevalence of NSSI, and the status of hepatitis B vaccination were major parameters used in the questionnaire.

### 2.5 Statistical analysis

Collected primary data were later tabulated and analyzed using cross‐tabulation and descriptive analysis. All the collected information was entered using Microsoft Excel and analyzed using the statistical package for social sciences version 22 and STATA version 15 for descriptive analysis. The normality of data was tested using the Shapiro–Wilk test. A *χ* ^2^ test was done to see the association between categorical variables and NSSI experience. Further association is delineated using logistic regression analysis using STATA for NSSI. Analyzed data were thus presented in the form of tables and charts and interpreted accordingly. The association is considered significant if the *p* < 0.05.

## 3 RESULTS

A total of 506 medical/nursing students and care providers were included in the study. The majority (64%) were females. Only about one‐third (29.6%) was older than 30 years. A total of 30.6% were doctors, followed by nursing students (29.6%) and nurses (20%), then medical students and paramedics. A total of 88.7% of the participants know having HBV risk (Table 1). NSSI was reported in 304 (60.08%), of them 19 (3.75%) reported being exposed >10 times (Supporting Information: Figure 1).

**Table 1:** Basic sociodemographic information of the study participants.

Variables	Frequency	Percentage
Profession		
Doctors	155	30.6
Nursing students	150	29.6
Nurses	101	20
Final‐year medical students	57	11.3
Paramedics	43	8.5
Gender		
Female	324	64
Male	182	36
Age group (In years)		
15–20	72	14.2
20–25	159	31.4
25–30	125	24.7
30+	150	29.6
Nationality		
Nepali	484	95.7
Indian	22	4.3
Job having exposure to HBV risk		
No	57	11.3
Yes	449	88.7
Risk status		
High	378	74.7
Low	80	15.8
Negligible	48	9.5

Among 304 injured participants, the maximum was exposed to blood percutaneously and most of the injury was due to hollow bore needles (46.1%) followed by suture needles. There was a slight difference in whether they know the HBV status of patients they were exposed to. IV cannulation was the maximum procedure and the emergency department (ER) was found at the major site for injury followed by the ward (Table 2). Nursing students were found to have exposure to NSSI with surprisingly lowest exposure experience in the laboratory (Supporting Information: Figure 2).

**Table 2:** Prevalence of NSSI with variables.

Variables	Frequency	Percentage
HBV status of exposed patient		
No	153	50.3
Yes	151	49.7
Exposure route		
Intramuscular	52	17
Mucous membrane	4	1
Nonintact skin	17	6
Percutaneous	231	76
Types of instruments involved		
Suture needle		
No	209	68.8
Yes	95	31.2
Scalpel		
No	282	92.8
Yes	22	7.2
Glass		
No	264	86.8
Yes	40	13.2
Hollow bore needle		
No	164	53.9
Yes	140	46.1
Blade		
No	267	87.8
Yes	37	12.2
Blunt object		
No	299	98.3
Yes	5	1.7
Other		
No	266	87.5
Yes	38	12.5
The site of injury occurred		
OPD		
No	294	96.7
Yes	10	3.3
Ward		
No	179	58.8
Yes	125	41.2
ER		
No	153	50.3
Yes	151	49.7
OT		
No	254	83.5
Yes	50	16.5
What procedure were you doing?		
Suture		
No	176	57.9
Yes	128	42.1
Operation		
No	272	89.5
Yes	32	10.5
IV cannulation		
No	149	49
Yes	155	51
Dressing		
No	222	73
Yes	83	27
Blood draw		
No	211	69.4
Yes	95	30.6
Others		
No	224	73.7
Yes	80	26.3
Body product/material you got exposed		
Blood		
No	146	48
Yes	158	52
Bloody fluid		
No	240	78.9
Yes	64	21.1
CSF		
No	294	96.7
Yes	10	3.3
Pleural fluid		
No	298	98
Yes	6	2
Peritoneal fluid		
No	296	97.3
Yes	8	2.7
Pericardial fluid		
No	304	100
Yes	0	0
Amniotic fluid		
No	287	94.4
Yes	17	5.6
Semen		
No	303	99.6
Yes	1	0.4
Vaginal fluid		
No	290	95.4
Yes	14	4.6

Abbreviations: CSF, cerebrospinalfluid; ER, emergency department; HBV, hepatitis B virus; IV, intravenous; NSSI, needle sticks and sharp‐related injuries; OPD, out patient department; OT, operation theatre.

**Figure 2:**
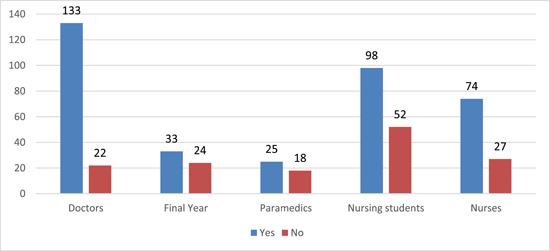
Hepatitis B virus vaccination among health professions.

Our participants believed most of the time NSSI happened accidentally and while not taking safety precautions. Few of them supported vision problems and lack of proper sleep to be the cause. Phobia and overconfidence in the procedure were least likely (Supporting Information: Figure 3). About 71.7% of total participants were found to be vaccinated against HBV and among them, 61.9% (44.5% of total HCWs included) have a total of three complete doses. Out of 363 vaccinated participants, only 34.2% of them had taken a booster dose (Supporting Information: Table 1). Among all the professionals, 133 doctors were found to have been vaccinated followed by nursing students (Figure 2).

While accessing the knowledge about the correct dose of the HBV vaccine that provides immunity in the body, the majority of the participants were unaware of it (Supporting Information: Figure 4).

The normality of data distribution was tested using the Shapiro–Wilk test. Our data were nonnormal in distribution. The *χ* ^2^ test was used to see the association between NSSI experience and determinant variables (profession, sex, age, vaccine status, and dose of vaccination). Among all tested variables, *p* < 0.05, suggesting a significant association (Table 3).

**Table 3:** Variables associated with NSSI.

Variables	NSSI experience		Total	*p* Value for *χ* ^2^
	No	Yes		
Profession				
Doctors	54 (10.7%)	101 (20%)	155 (30.6%)	<0.001
Final year	50 (9.9%)	7 (1.4%)	57 (11.3%)	
Nurses	27 (5.3%)	74 (14.6%)	101 (20%)	
Nursing students	47 (9.3%)	103 (20.4%)	150 (29.6%)	
Paramedics	24 (4.7%)	19 (3.8%)	43 (8.5%)	
Total	202 (39.9%)	304 (60.1%)	506 (100%)	
Sex				
Female	109 (21.5%)	215 (42.5%)	324 (64%)	<0.001
Male	93 (18.4%)	89 (17.6%)	182 (36%)	
Age				
15–20	20 (4%)	52 (10.3%)	72 (14.2%)	<0.001
20–25	86 (17%)	73 (14.4%)	159 (31.4%)	
25–30	41 (8.1%)	84 (16.6%)	125 (24.7%)	
30+	55 (10.9%)	95 (18.8%)	150 (29.6%)	
Vaccine				
No	70 (13.8%)	73 (14.4%)	143 (28.3%)	<0.05
Yes	132 (26.1%)	231 (45.7%)	363 (71.7%)	
Dose				
0	70 (13.8%)	73 (14.4%)	143 (28.3%)	<0.05
1	6 (1.2%)	18 (3.6%)	24 (4.7%)	
2	33 (6.5%)	81 (16%)	114 (22.5%)	
3	93 (18.4%)	132 (26.1%)	225 (44.5%)	

Abbreviation: NSSI, needle sticks and sharp‐related injuries.

Further, the association was delineated using multiple logistic regression for the occurrence of NSSI experience as 1 and no such experience as 0. Multiple logistic regression analysis of tested variables showed less NSSI experience with AHCW (aOR: 0.4) and clinical year medical students (aOR: 0.08) in reference to nurses. However vaccinated individuals have higher odds of having NSSI experience (aOR: 4.5). Though age and sex categories were found to have associated in the *χ* ^2^ test, multiple logistic regression analysis adjusting with other tested variables did not show such association (Supporting Information: Table 2).

## 4 DISCUSSION

Among 506 participants in our study, the majority were females (64%), with age groups ranging from 15 to 30 years of age. Most of the participants were in the age group of 20–25 years. Doctors (30.6%) were the major profession followed by nursing students (29.6%) among the participants. Among participants, 88.7% were aware of being at the high risk to the HBV exposure. In our current study, we found that 304 (60.08%) reported being exposed to NSSI. This finding is consistent with similar studies conducted among nursing and midwifery students in Eastern Ethiopia, where 62.8% reported NSSI.^12^ We also observed a comparable rate among nurses working in Northeast Ethiopia, with 34.5% self‐reporting needle stick injuries. Notably nursing students accounted for the higher proportion of injuries in our study (35.5%).^13^ On the other hand, our finding was lower than a study conducted among midwives and nurses in Northeast Ethiopia, where 75.5% reported NSSI.^14^ Additionally, the NSSI injury rate in our study was higher than a similar study conducted among HCWs in Nepal, which reported a prevalence of 37.6% of Needle stick injuries.^15^ Another study carried out in Nepal among HCWs reported an NSSI rate of 74%.^16^ In our study, NSSI was more prevalent among individuals aged >30, whereas a study conducted at B.P. Koirala Institute of Health Sciences (BPKIHS), Nepal, found that NSSI was more common in the ≤22 years age group.^8^

A significant association was found between the prevalence of NSSI and the types of profession (*p* < 0.001) which has been supported by the studies done in other parts of Nepal (*p* < 0.05).^15^ Our study also showed a significant difference between the prevalence of NSSI and other variables like age, sex, vaccination against HBV, and so on. This finding is consistent with studies done by Bhattrai et al.^10^ Similarly, Knowledge was assessed for the prevalence of NSSI with Sex and was found to have no any significant differences.^9^ However, in the study done among HCWs of Saudi Arabia, no any evidence of an association between NSSI with profession and sex had been found.^4^

In the study done at students of eastern Ethiopia, males were 1.56 times more likely to be injured with NSSI while in our study after analyzing with multiple logistic regression, no significant association was seen between sex and age.^12^ Our study showed NSSI experience was low in ACHWs and clinical‐year medical students in comparison to the nurses. In contrast to our study, NSSI was not found to be significantly associated with the Nurses done in the study in Northeast Ethiopia.^14^

The majority of incidents in our study occurred with hollow bore needles, consistent with other studies conducted in Nepal.^6^ Mon et al., 2014 in their study report the same finding.^17^ The most common place of injury was the ER (49.7%) followed by the ward. This could be attributed to rush hours and the increased need for sample collection in the ER. About 52% of the participants had been accidentally exposed to the blood which aligns with a study conducted among medical students in Cameroon.^18^ Regarding hepatitis B vaccination status, our study found a vaccination rate of 71.7% among HCWs, which is slightly lower than a study conducted among medical students at BPKIHS, where 86.5% were vaccinated. This difference could be attributed to the inclusion of a broader range of HCWs in our study, such as doctors, nurses, nursing students, medical students, health assistants, and laboratory staff from NGMCTH. Similarly, a study conducted at Chitwan medical college, reported a vaccination rate of 73.5% among preclinical and dental students, which is comparable to our findings.^7^, ^10^ Several studies have highlighted a low rate of complete vaccination among medical students, as seen in a study by Chhabra et al., where 82.8% of students‐initiated vaccination, but only 62.4% completed the recommended three doses. In our study, 61.98% of the vaccinated participants received the full three doses.^19^ In the study conducted at Manipal Medical College, only 47.1% had completed all three doses which is slightly lesser than our study.^8^ Most cases of HBV infection resulting from NSSI are due to accidental percutaneous exposure, which is supported by our study findings. Therefore, it is essential for HCWs to be mindful of preventive measures.^20^ In our study, only 2.4% of respondents hecked their immune status postvaccination, which is lower compared to studies by Bhattarai et al. (9.3%) and Chhabra et al. (7%), where participants measured their antibody titer.^8^, ^19^

## 5 CONCLUSION

HCWs are exposed to accidental injuries despite taking necessary precautions. There is a risk of adverse health effects for HCWs on being exposed to NSSI. Enough academic training to avoid these injuries must be advocated. There should be a standardized procedure with universal precautions to be taken for handling blood and body fluids. Guidelines should be implemented for early and immediate serology investigations before carrying out any procedures with any sharp objects. All needle stick injuries must be reported postexposure. There should be a standard protocol for postexposure counseling and treatment. A better surveillance system for registration, reporting, and management of such hazards must be established at every hospital. In addition, hepatitis B immunization programs for HCWs at risk of exposure should be implemented by all health facilities and the government with a high level of motivation.

## AUTHOR CONTRIBUTIONS

**K. C. Rupak**: Conceptualization; data curation; writing—original draft; writing—review and editing. **Dipendra Khadka**: Conceptualization; supervision. **Sabal Ghimire**: Data curation; methodology; resources; writing—original draft; writing—review and editing. **Aayush Bist**: Investigation; resources; writing—original draft; writing—review and editing. **Ishant Patel**: Data curation; writing—original draft. **Smriti Shahi**: Data curation; writing—original draft; writing—review and editing. **Natasha Dhakal**: Data curation; project administration; resources; writing—review and editing. **Ibeja Tiwari**: Data curation; project administration; writing—review and editing. **Dhan B. Shrestha**: Conceptualization; formal analysis; methodology; software; supervision; writing—review and editing.

## CONFLICT OF INTEREST STATEMENT

The authors declare no conflict of interest.

## ETHICS STATEMENT

Ethics approval for the research project was obtained from the NGMCTH Ethics Review Committee. All co‐authors have read and approved the final version of the manuscript. The lead author K. C. Rupak had full access to all of the data in this study and takes complete responsibility for the integrity of the data and the accuracy of the data analysis.

## TRANSPARENCY STATEMENT

The lead author K. C. Rupak affirms that this manuscript is an honest, accurate, and transparent account of the study being reported; that no important aspects of the study have been omitted; and that any discrepancies from the study as planned (and, if relevant, registered) have been explained.

## Supporting information

Supporting information.

## Data Availability

The authors confirm that the data supporting the findings of this study are available within the article and its supplementary materials.
