# Supplementary material for: Prevalence of exposure to needle stick and sharp‐related injury and status of hepatitis B vaccination among healthcare workers: A cross‐sectional study
Source: Health Sci Rep. 2023 Jul 5;6(7):e1399. doi: 10.1002/hsr2.1399 (PMC10320746; doi:10.1002/hsr2.1399)
Supplement: Supplementary file 1 — Supporting information. [file HSR2-6-e1399-s001.docx]

**Prevalence of exposure to needle stick and sharp related injury and status of hepatitis B vaccination among health care workers: A cross-sectional Study**

**Figure 1:** Frequencies of needle sticks and sharp-related injuries among NSSI-experienced individuals.

**Figure 2**: Department’s Exposure to Injuries.

**Figure 3:**  Opinions regarding causes of NSSI injury

**Table 1**: HBV vaccination status among participants.

| Variables | | Frequency | Percentage |
| --- | --- | --- | --- |
| Vaccine | No | 143 | 28.3 |
|  | Yes | 363 | 71.7 |
| Among 363 participants who were Vaccinated | 1^st^ dose | 24 | 6.6 |
|  | 2^nd^ dose | 114 | 31.4 |
|  | 3^rd^ dose | 225 | 61.9 |
| Booster Dose among vaccinated | Yes | 124 | 34.2 |
|  | No | 239 | 65.8 |

**Figure 4**: Knowledge about Dose providing immunity against HBV.

**Table 2**: Multiple logistic regression analysis of tested variable for the occurrence of NSSI

| NSSI experience | Adjusted Odds Ratio | Std. Err. | z | P>\|z\| | [95% Conf. Interval] | |
| --- | --- | --- | --- | --- | --- | --- |
| Profession | Nurse® | | | | | |
| AHCW | .3953705 | .1674776 | -2.19 | 0.028 | .1723607 | .9069227 |
| Doctors | .9627624 | .3614544 | -0.10 | 0.919 | .461263 | 2.009508 |
| Nursing students | .629474 | .2381719 | -1.22 | 0.221 | .2998553 | 1.321429 |
| Clinical year MBBS | .0757221 | .0421609 | -4.63 | 0.000 | .0254265 | .2255064 |
| Sex | Female® | | | | | |
| Male | .6319243 | .1966904 | -1.47 | 0.140 | .3433391 | 1.163073 |
| Age categories | 15-20® | | | | | |
| 20-25 | .5526822 | .2059441 | -1.59 | 0.112 | .2662534 | 1.147244 |
| 25-30 | .7524018 | .3213025 | -0.67 | 0.505 | .3258039 | 1.737574 |
| 30+ | .543063 | .2503543 | -1.32 | 0.185 | .2200102 | 1.340472 |
| Vaccination | No® | | | | | |
| Yes | 4.552008 | 2.62988 | 2.62 | 0.009 | 1.466997 | 14.12462 |
| Dose | .6659765 | .1376098 | -1.97 | 0.049 | .4441977 | .9984849 |
| Constant | 3.240214 | 1.484839 | 2.57 | 0.010 | 1.319798 | 7.954992 |
